# Supplementary material for: Clinical impact of pathology-proven etiology of severely stenotic aortic valves on mid-term outcomes in patients undergoing surgical aortic valve replacement
Source: PLoS One. 2020 Mar 10;15(3):e0229721. doi: 10.1371/journal.pone.0229721 (PMC7064191; doi:10.1371/journal.pone.0229721)
Supplement: Attachment — Submitted filename: Final Response to Reviewers.docx [file pone.0229721.s003.docx]

**Response to Reviewer’s comments**

**Their revision and written communication addresses some of the comments/questions raised by reviewers. They are careful to note their association between valve type is not causal. The clinical impact of this investigation is difficult to ascertain. The authors might include additional discussion on their interpretation of results – i.e. their interpretation of findings. They may have an idea on why, after risk adjustment, patients with calcific/degenerative pathology have worse outcomes (fatal and nonfatal complications) relative to patients with inflammatory pathology - especially given the strong association seen with adverse outcomes**

Response: Because of potential residual confounding, we agree with the reviewer that the association which we found are most likely not fully causal. We do like to discuss potential reasons for the association. Based on our findings, we speculate that the valve etiology profiles might have some effect on mid-term outcomes even after the removal of the diseased valve. Possibly, those differences in late complications among three groups may stem from the extent of postoperative regression of diffuse myocardial fibrosis and myocardial cellular hypertrophy based on aortic valve etiology, as investigated in a recent study [1]. Recently, calcific and degenerative aortic stenosis (AS) has been understood to be an active disease process akin to atherosclerosis from the perspectives of compelling histopathologic and clinical data [2]. Our study results allow us to interpret that numerous overlaps in clinical factors related to calcific and degenerative valve disease and atherosclerosis might be a key finding to explain the potential mechanism by which patients in the calcific/degenerative group develop adverse events associated with plaque vulnerability and thrombosis, including new-onset ischemic heart disease and non-fatal stroke, even after surgical aortic valve replacement (AVR) with concomitant coronary artery bypass grafting (CABG) when indicated. Indeed, heart failure admission, unplanned coronary revascularization and cardiovascular death were more common in the calcific and degenerative compared to the other two groups. The systemic impact of these atherosclerotic disease may in part be considered a residual confounding when comparing the prognoses of surgical AVR patients with three different etiologies. However, it can also be considered a direct consequence of the AS that differently affects myocardium and its recovery by AS etiology. In contrast, congenital aortic valve disease often involves many vascular abnormalities [3], and post-inflammatory disease can affect the myocardium and heart valves and the brain, joints, and skin [4]. In our present study, 23% of patients in the congenital group had undergone replacement of ascending aorta concomitantly, and 82% and 36% of patients in the post-inflammatory group were added surgical procedures on mitral valve and tricuspid valve, respectively. These concomitant surgical procedures, when indicated, might show protective effect on clinical outcomes for potential late complications after surgical AVR. In other words, other valve abnormalities and aortic disease (aneurysm and dilatation) are more likely to coexist in both post-inflammatory and congenital AS patients, which could raise a risk of developing cardiovascular events. However, in most cases, these cardiac comorbidities are surgically treated concomitantly when indicated based on current guidelines, leading to risk reduction for potential cardiovascular events to occur after surgical AVR. On the other hand, patients with calcific/degenerative AS appear to have a strong association with systemic atherosclerosis and there might be an aspect that cardiovascular risks are inevitably left even though a concomitant CABG is properly performed.

Thus, we have strengthened the discussion on why patients with calcific/degenerative pathology have worse outcomes (fatal and nonfatal complications) relative to patients with inflammatory pathology (Page 26, Line 347): “Based on our findings, we speculate that the valve etiology profiles might have some effects on the mid-term outcomes even after the removal of the diseased valve. Possibly, those differences in late complications among the three groups may stem from the extent of postoperative regression of diffuse myocardial fibrosis and myocardial cellular hypertrophy based on the aortic valve etiology, as investigated in a recent study [27]. Recently, calcific and degenerative AS has been understood to be an active disease process akin to atherosclerosis from the perspectives of compelling histopathologic and clinical data [28]. Our study results allow us to interpret that numerous overlaps in clinical factors related to calcific and degenerative valve disease and atherosclerosis might be a key finding to explain the potential mechanism by which patients in the calcific/degenerative group develop adverse events associated with plaque vulnerability and thrombosis, including new-onset ischemic heart disease and non-fatal stroke, even after surgical AVR with concomitant CABG when indicated. Indeed, HF admission, unplanned coronary revascularization, and cardiovascular death were more common in the calcific and degenerative group than in the other two groups. The systemic impact of these atherosclerotic diseases may in part be considered a residual confounding when comparing the prognoses of surgical AVR patients with three different etiologies. However, it can also be considered a direct consequence of the AS that differently affects the myocardium and its recovery by the AS etiology. In contrast, the congenital aortic valve disease often involves many vascular abnormalities [29], and the post-inflammatory disease can affect the myocardium and heart valves and the brain, joints, and skin [30]. In our present study, 23% of patients in the congenital group had undergone replacement of ascending aorta concomitantly, and 82% and 36% of the patients in the post-inflammatory group underwent additional surgical procedures on the mitral and tricuspid valves, respectively. These concomitant surgical procedures, if indicated, might show a protective effect on the clinical outcomes for potential late complications after surgical AVR. In other words, other valve abnormalities and aortic diseases (aneurysm and dilatation) are more likely to coexist in both post-inflammatory and congenital AS patients, which could raise a risk of developing cardiovascular events. However, in most cases, these cardiac comorbidities are surgically treated concomitantly when indicated based on the current guidelines [7], leading to risk reduction of potential cardiovascular events that could occur after surgical AVR. On the other hand, patients with calcific/degenerative AS appear to have a strong association with systemic atherosclerosis, and there might be a possibility that cardiovascular risks inevitably remain even though a concomitant CABG is properly performed. Taking this into consideration, AS should be deemed as a systemic disease with different potential mechanisms by its valve etiology, although the definite pathophysiology behind AS remains incompletely investigated [31].”

**Consider including the results of univariate analysis for the candidate variables use for multivariate analysis used in Table 4 (i.e.,, age, sex,....euroscore, etc)**

Response: We have added the results of univariate analysis for all variables that were used for each multivariate analysis changing the format of Table 4.

**Also, In the discussion, the authors might briefly discuss recommendations on how their study findings should impact clinical practice.**

Response: Our data suggest that the etiology and pathobiology of the valvular disease should be examined through a pathological investigation of the excised valve, which can provide essential information on its natural history, surgical risk, postoperative outcome, association with systemic disease, and potential vascular complications, in addition to preoperative imaging assessments. Therefore, future possibility of establishing accurate diagnosis of the diseased valves with less invasive imaging modalities confirmed by pathological examinations should be further explored, which can lead to another novel researches such as valve progression by aortic valve etiology or associations with non-aortic valves. In addition, our results show that the pathologically assessed etiology of the diseased valve was a strong predictor of the mid-term outcomes of the patient even after adjusting for patient demographic characteristics and perioperative risks (represented by EuroSCORE II). This could certainly guide clinicians and patients in decision-making regarding the postoperative follow-ups and support the importance of the accuracy of pathological valve diagnosis. More importantly, our data on detailed clinical outcomes highlights distinct trends in incidences of harmful events following surgical AVR among three etiologies. In fact, heart failure admission, device implantation and gastrointestinal bleeding were more prominent in both the congenital and calcific/degenerative groups compared to the post-inflammatory one while non-fatal stroke was more common in both the calcific/degenerative and post-inflammatory groups. In managing these patients, such a novel information could help to detect and treat potential late complications unique to each valve etiology.

Thus, we reinforced the discussion section to clearly state clinical implications (Page 27, Line 382): “As a clinical implication, our data suggest that the etiology and pathobiology of the valvular disease should be examined through a pathological investigation of the excised valve, which can provide essential information on its natural history, surgical risk, postoperative outcome, association with systemic disease, and potential vascular complications, regardless of preoperative imaging assessments. Therefore, future possibility of establishing accurate diagnosis of the diseased valves with less invasive imaging modalities confirmed by pathological examinations should be further explored, which can lead to other novel researches, such as valve progression by aortic valve etiology or associations with non-aortic valves. Moreover, our data on the detailed clinical outcomes highlight distinct trends in the incidences of harmful events following surgical AVR among the three etiologies. For instance, HF admission, device implantation, and gastrointestinal bleeding were more prominent in both the congenital and calcific/degenerative groups than in the post-inflammatory group, whereas non-fatal stroke was more common in both the calcific/degenerative and post-inflammatory groups. In managing these patients, such novel information could help to detect and treat potential late complications unique to each valve etiology.”

**Figures 3 and 4 are difficult to read. Consider reformatting the figures so the graphs could be enlarged.**

Response: We have reformatted the figures 3 and 4 so that they could be more readable by enlarging the original graphs clearly.

**Reference**

1.	Treibel TA, Kozor R, Schofield R, Benedetti G, Fontana M, Bhuva AN, et al. Reverse Myocardial Remodeling Following Valve Replacement in Patients With Aortic Stenosis. J Am Coll Cardiol. 2018;71:860-71. doi: 10.1016/j.jacc.2017.12.035 PMID: 29471937

2.	Freeman RV, Otto CM. Spectrum of calcific aortic valve disease: pathogenesis, disease progression, and treatment strategies. Circulation. 2005;111:3316-26. doi: 10.1161/CIRCULATIONAHA.104.486738 PMID: 15967862.

3.	Siu SC, Silversides CK. Bicuspid aortic valve disease. J Am Coll Cardiol. 2010;55:2789-800. doi: 10.1016/j.jacc.2009.12.068 PMID: 20579534.

4.	Marijon E, Mirabel M, Celermajer DS, Jouven X. Rheumatic heart disease. Lancet. 2012;379:953-64. doi: 10.1016/S0140-6736(11)61171-9 PMID: 22405798.
