# Supplementary material for: Clinical impact of pathology-proven etiology of severely stenotic aortic valves on mid-term outcomes in patients undergoing surgical aortic valve replacement
Source: PLoS One. 2020 Mar 10;15(3):e0229721. doi: 10.1371/journal.pone.0229721 (PMC7064191; doi:10.1371/journal.pone.0229721)
Supplement: Attachment — Submitted filename: Response to Academic Editor.docx [file pone.0229721.s004.docx]

**Response to Reviewer’s comments**

**The manuscript is improved and the reviewer's comments were addressed. However, the current discussion is too lengthy, with run-on sentences, redundant ideas and unnecessary repetition of the results. Suggest to summarize the key results along with the main discussion points in a concise manner. Limit the reviews of the topic to only what is pertinent to the discussion points. Please also review the final product with a professional English editor.**

Response: As suggested by academic editors, we carefully summarized the main discussion points for improved readability and clarity and fully focused on the topic of our interests throughout the entire manuscript by avoiding redundancy and unnecessary ideas. Thus, we revised some parts, mainly in the discussion section, with our intended meanings retained that are highlighted in red ink so that you could follow the changes we made. This file was uploaded as a separate file and labeled 'Revised Manuscript with Tracked Changes' together with ‘Manuscript’ in the process of submitting the revised manuscript after the final product was carefully reviewed by a professional English editor.
